# Practitioner’s knowledge, attitudes, beliefs and practices towards urinary incontinence

**DOI:** 10.4102/sajp.v79i1.1860

**Published:** 2023-06-30

**Authors:** Anika C. Janse van Vuuren, Jacobus A. van Rensburg, Susan Hanekom

**Affiliations:** 1 Department of Physiotherapy, Faculty of Health Sciences, Stellenbosch University, Cape Town, South Africa; 2 Department of Obstetrics and Gynaecology, Urogynaecology Unit, Tygerberg Hospital and Stellenbosch University, Cape Town, South Africa

**Keywords:** attitude, belief, knowledge, practice, primary healthcare, urinary incontinence

## Abstract

**Background:**

One in three women in South Africa suffer from urinary incontinence. Effective management is influenced by patients help-seeking behaviour and services offered by healthcare professionals within the healthcare system. Current practice towards urinary incontinence management in South Africa is unknown.

**Objectives:**

Our study aimed to describe and compare urinary incontinence practice and knowledge of nurses and physicians (practitioners) working in primary healthcare settings, measured against the NICE 2013 guideline and explore attitudes and beliefs towards urinary incontinence management.

**Method:**

Cross-sectional study using a self-designed online questionnaire. All primary healthcare practitioners in the Western Cape were eligible for the study. Stratified random and snowball sampling was used. Data was analysed in consultation with a statistician using SPSS.

**Results:**

Fifty-six completed questionnaires were analysed. Practitioners had an overall knowledge score of 66.7% and practice score of 68.9% compared to NICE 2013 guidelines. A lack of knowledge regarding urinary incontinence screening, following up on patients and conducting bladder diaries were noted. Pelvic floor muscle training and bladder training education was recognised as initial management but only 14.8% of practitioners referred patients to physiotherapy. Half of the sample reported being uncomfortable with urinary incontinence, although the majority wanted to learn more about urinary incontinence.

**Conclusion:**

The knowledge and practices of practitioners working at a primary healthcare level in the Western Cape are not congruent with NICE 2013 guidelines.

**Clinical implications:**

Data can be used to inform intervention planning to address urinary incontinence management at a primary healthcare level in the Western Cape.

## Introduction

In South Africa, urinary incontinence (UI) affects roughly one-third of the female population (Bailey et al. 2010; Jacobs, Hanekom & Rensburg 2017; Madombwe & Knight 2010; Skaal & Mashola 2011) ranging from 27.5% to 35.4%. A recent meta-analysis found that one in five women in sub-Saharan Africa presents with UI (Ackah et al. 2022). The physical and psychological effects of UI on patients’ health-related quality of life (HRQOL) have been well described. Urinary incontinence impairs activities of daily living in patients (Norton 2007). The social and cultural stigma attached to UI affects patients’ perceptions of their HRQOL (Norton 2007), and minimal UI is associated with significant negative impacts on HRQOL. However, the economic burden of UI has primarily been described in the developed world, with limited economic data available from Africa (Milsom et al. 2014).

The current evidence for effective conservative management of UI, including physiotherapy, is convincing (National Institute for Health and Care Excellence [NICE] 2013; Vaz et al. 2019). Various practice management guidelines have been developed to guide decision-making (Heyns & Rienhardt 2002; NICE 2013; Vaz et al. 2019). The NICE (2013) guideline scored 97% on the Appraisal of Guidelines for Research and Evaluation score compared to other guidelines (Bravo-Balado et al. 2019) and is recommended for use. The South African guideline for UI management was developed in 2002 and did not include current evidence (Heyns & Rienhardt 2002). Despite the availability of high-quality evidence and practice guidelines, the poor health-seeking behaviour of women suffering from UI and limited knowledge of healthcare practitioners (HCPs) at a primary healthcare (PHC) level prevent optimal management. Community awareness, as well as public and HCPs education, is required to facilitate primary prevention (Norton 2007). It has been argued that it is the HCP’s responsibility to routinely enquire about UI (Norton 2007).

High-level evidence suggests that early detection and management of UI by HCPs at a PHC level can affect the morbidity associated with UI and be a cost-effective strategy to manage UI (Holtzer-Goor et al. 2015). The early referral of patients to physiotherapy by HCPs at a PHC level is hypothesised to further reduce costs (Vaz et al. 2019).

Various factors impact the clinical practice of all HCPs. Factors include knowledge, attitudes, beliefs and practices towards UI (Park et al. 2015) and the characteristics of the social network and organisational, financial and structural aspects of the environment in the HCPs work (Albers-Heitner et al. 2008). Further, the implementation model of Grol identifies HCPs’ competencies, attitude, motivation for change and personal characteristics as important aspects to understand before implementing evidence-based practice (EBP) (Albers-Heitner et al. 2008).

We previously conducted a scoping review to describe which HCPs have been investigated and how HCPs’ knowledge, attitudes, beliefs and practices towards UI management have been explored (Van Vuuren et al. 2021). Studies from our scoping review highlighted that the knowledge, attitudes, beliefs and practices differed between HCPs, settings and countries. While we did identify a number of studies that were conducted in low-resource settings such as in Thailand (Sarit-Apirak, Udomsubpayakul & Manonai 2016), India (Sinha et al. 2018) and Brazil (Tomasi et al. 2017), we found only one study reporting conditions in South Africa. The study explored South African general practitioners’ (GPs’) knowledge, attitudes and practices regarding female patients with UI (Padayachey 2009). Physicians and nursing practitioners are the first contacts of all patients visiting PHC clinics. To strengthen the knowledge base from South Africa and inform the planning of future interventions which could facilitate optimal UI management in South Africa, the aims of our study are to report on the knowledge, attitudes and beliefs of primary physicians and nursing practitioners towards UI management and to describe current UI management at PHC facilities in the Western Cape.

## Methods

A cross-sectional study was conducted using a self-designed online questionnaire. The questionnaire was developed based on the NICE: *Urinary Incontinence in Women: The Management of Urinary Incontinence in Women* (NICE 2013) guideline along with algorithms from Abrams et al. (eds. 2017) relating to the initial management of UI in women. Results from a scoping review (Van Vuuren et al. 2021) were used to further refine the questionnaire and determine the aspects of attitudes and beliefs towards UI management to be explored. The questionnaire consisted of six sections: facility, demographics, PHC practitioner demographics, UI screening and evaluation, UI management and referral of UI patients.

The questionnaire was piloted (Online Appendix 1 – Addendum A–E) to determine if the questions were interpreted correctly and the time required for completion. The final questionnaire was adjusted by changing the layout to make the questionnaire more user-friendly, and questions were altered to make them clearer (Online Appendix 1 – Addendum F). Thereafter, the questionnaire was translated from English to Afrikaans (Online Appendix 1 – Addendum G) by the first author and co-authors, as well as to isiXhosa (Online Appendix 1 – Addendum H) by Stellenbosch University’s language department, as these are the predominant languages in the Western Cape.

Questions on UI management practices were based on NICE 2013 guidelines (NICE 2013). Taking into account the hypothesis that there is a 50% adherence of practice to the guideline with a 5% confidence interval, an estimated sample size of 384 HCPs was calculated.

Two sampling strategies were used. Firstly, we randomly selected Western Cape healthcare facilities stratified for level of care in each municipality. The Western Cape is divided into six subdistricts, namely, West Coast, Cape Winelands, Overberg, Garden Route, Central Karoo and Cape Metropole. These subdistricts are further divided into local municipalities. Microsoft Excel was used to conduct a stratified random sampling approach to select one clinic in each municipality. Forty-three facilities were selected. Unfortunately, as a result of adaptations to the services provided at PHC clinics because of coronavirus disease 2019 (COVID-19) management, the Department of Health only allowed us to contact 17 facilities. A list of all included facilities is shown in Online Appendix 1 – Addendum L. Once a facility agreed to participate, the Sister in charge distributed a questionnaire link to relevant facility staff. Secondly, in an endeavour to reach the estimated sample size, we employed a snowball sampling technique, using two primary data sources: (1) family physicians associated with family physician forums run by Stellenbosch and Cape Town University and (2) associates of the Stellenbosch 2016 Bachelor of Medicine and Bachelor of Surgery (MBChB) class. The invitation requested recipients to further distribute the questionnaire to PHC medical practitioners and nursing practitioners in the Western Cape.

Two strategies were used to increase participation: (1) primary contacts identified from the data sources used in the snow-ball sampling technique were emailed three times by the first author, over a 3-week period as a reminder to distribute questionnaires; and (2) an online continuous professional development accredited activity ‘Initial UI management at a PHC level’ was developed by the first author in collaboration with experts in the field and made available to participants after completion of the survey (Online Appendix 1 – Addendum K).

All medical practitioners and nursing practitioners working in a Western Cape PHC facility and who provided informed consent were included in our study.

Data analysis was conducted by the first author using Excel and Statistical Package for the Social Sciences version 23 (SPSS, IBM Corp. Released 2020. IBM SPSS Statistics for Windows, Version 27.0. Armonk, New York, United States, with guidance from a statistician. Descriptive statistics were calculated. Categorical data were summarised as frequencies and percentages. Continuous data were summarised using means and standard deviations, as well as medians and interquartile ranges. Ninety-five per cent confidence intervals (CI) were used where appropriate. Parametric and non-parametric tests were used based on data distribution. For correlation analyses, Pearson correlation tests were used for normally distributed data, and Spearman’s rho correlation tests were used for non-normally distributed data. Correlation relationship strength was categorised as no relationship (*r* < 0.25), weak (0.25 < *r* < 0.5), moderate (0.5 < *r* < 0.75) and strong (*r* > 0.75). Student’s *t*-tests were used to compare HCPs groups for continuous variables, and Chi-squared tests or Fisher’s exact tests were used for categorical variables (as appropriate). Significant differences between groups are reported at the alpha level of 0.05. All reported *p*-values are two-sided. Risk estimates were calculated with a cohort outcome ‘Yes’.

Knowledge and practice questions were based on the ‘gold standard’ NICE 2013 guidelines (NICE 2013), and therefore, answers could be scored on how closely guidelines were adhered to. The first author developed a memorandum a priori in order to score the questionnaire. Eighteen knowledge questions and 14 practice questions were included. Participants scored one point for each correct answer, allowing for an overall knowledge (total score 18) and practice (total score 14) score. Referral of failed initial UI management scored a point, regardless of who the HCP referred to, as it ensured patients were not lost in the system. Attitudes and beliefs could not be compared to a ‘gold standard’, and therefore, results are described descriptively.

### Ethical considerations

Ethics approval was obtained from the Health Research and Ethics Committee at Stellenbosch University (Online Appendix 1 – Addendum I) (S10/09/173). Further permission was obtained from the Western Cape Department of Health (Online Appendix 1 – Addendum J) to access PHC government facilities. All participants provided written informed consent in the language of choice. The questionnaire was available between April 2021 and August 2021.

## Results

**FIGURE 1:**
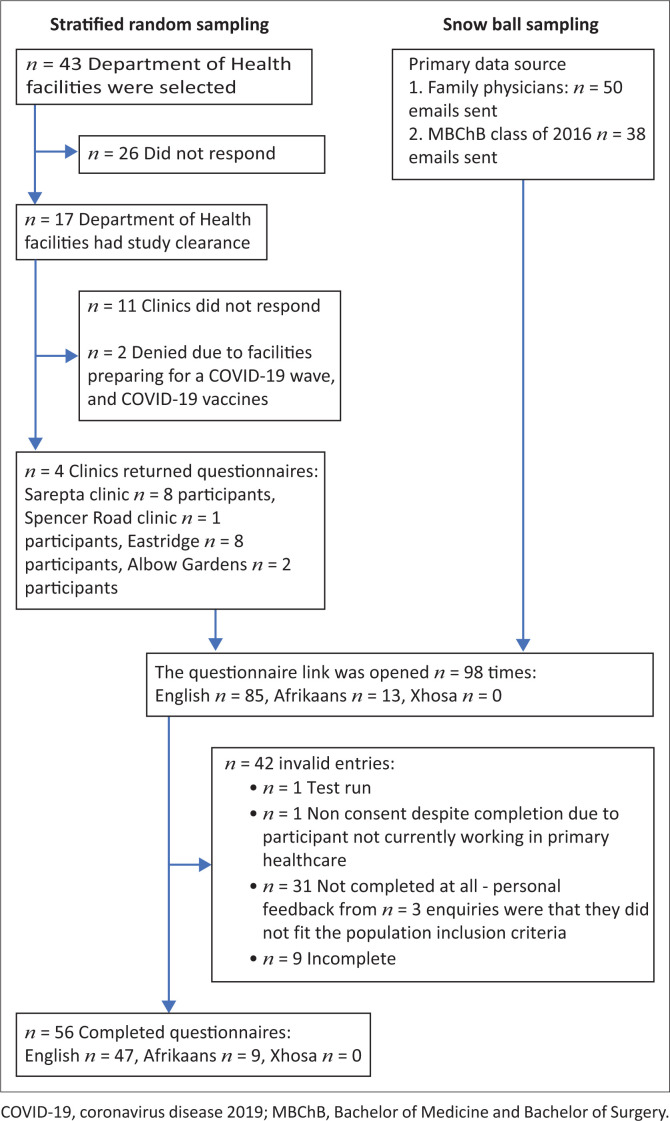
Participant flow diagram.

Fifty-six questionnaires were analysed (Figure 1). A response rate could not be determined, as it is unknown how many emails were received or forwarded to other PHC practitioners.

### Facility demographics

Data are predominantly from urban areas, with limited rural area representation. Healthcare practitioners indicated UI was managed at more than half (58.9% *n* = 33) of the facilities, and 66.1% (*n* = 37) of participants reported that there was a UI referral pathway at their facility. Eighty-two per cent (*n* = 46) of the facilities were located near a secondary or tertiary referral hospital, and 23.2% (*n* = 13) of the facilities had printed or computer access to UI guidelines. Healthcare practitioners elaborated on guidelines used at their facilities. Guidelines included the practical approach to care (PACK) guideline (*n* = 9), standard treatment guidelines – primary care level (*n* = 1), essential medical (EM) application (*n* = 4) and essential medicines list (EML) (*n* = 1). One participant stated that they follow a gynaecologist on SharePoint but did not disclose their reasoning. Regular general education sessions were available in 58.9% (*n* = 33) of facilities, although most facilities (92.9%, *n* = 52) reported that UI was not part of their education programmes. Educational sessions were available in the form of tutorials (57.6%, *n* = 19), reading material (45.4%, *n* = 15), practical demonstrations (30.3%, *n* = 10) and Zoom or TEAMS meetings (36.4%, *n* = 12).

### Participant demographics

Most responses were from the public sector (92.9%, *n* = 52), and 64.2% (*n* = 36) of respondents had an MBChB qualification. All nursing respondents and 61.1% (*n* = 22) of medical practitioners were based in the Cape Town metropole, providing three-quarters of the data (75%, *n* = 42). Healthcare practitioners predominantly worked in clinics (55.4%, *n* = 31) and community day centres (32.1%, *n* = 18). Most responses were female (83.9%, *n* = 47). Male participants arose from the medical practitioner population. Online Appendix 1 – Addendum N displays the population demographics of private and government HCPs (Table 1). No questionnaires were completed in Xhosa, despite being distributed to Xhosa ethnic groups. Healthcare practitioners indicated that despite being Xhosa, they were educated in English, and therefore, they prefer English when it comes to professional communication.

**TABLE 1:** Medical practitioner and nursing practitioner population demographics (*n* = 56).

HCPs demographics	Whole population (*n* = 56)						Medical practitioner (*n* = 36)						Nurses (*n* = 20)					
	Mean	s.d.	Median	IQR	*n*	%	Mean	s.d.	Median	IQR	*n*	%	Mean	s.d.	Median	IQR	*n*	%
**Age (years)**	38.0	9.6	38.0	15.0	-	-	36.0	8.5	37.0	14.5	-	-	42.0	10.6	43.0	14.5	-	-
**Gender**																		
Male	-	-	-	-	9	16.1	-	-	-	-	9	25.0	-	-	-	-	0	0.0
Female	-	-	-	-	47	83.9	-	-	-	-	27	75.0	-	-	-	-	20	100.0
**Job qualifications**																		
Medical practitioner (MBChB)	-	-	-	-	36	64.3	-	-	-	-	36	100.0	-	-	-	-	0	0.0
Advanced diploma in midwifery	-	-	-	-	0	0.0	-	-	-	-	0	0.0	-	-	-	-	0	0.0
Higher certificate auxiliary nursing qualification	-	-	-	-	3	5.4	-	-	-	-	0	0.0	-	-	-	-	3	15.0
Diploma in nursing: staff nurse	-	-	-	-	2	3.4	-	-	-	-	0	0.0	-	-	-	-	2	10.0
Bachelor’s degree in nursing and midwifery	-	-	-	-	8	14.3	-	-	-	-	0	0.0	-	-	-	-	8	40.0
Post graduate diploma in nursing or midwifery or accoucheur or primary care nursing	-	-	-	-	6	10.7	-	-	-	-	0	0.0	-	-	-	-	6	30.0
Not selected†	-	-	-	-	1	1.8	-	-	-	-	0	0.0	-	-	-	-	1	5.0
**Years in practice**	12.0	9.2	12.0	15.0	-	-	10.5	8.3	10.0	13.25	-	-	14.8	10.4	14.0	15.5	-	-
**Years at current facility**	4.7	5.6	2.0	6.0	-	-	4.1	4.4	2.0	6.0	-	-	6.0	7.2	3.3	6.8	-	-
**District municipalities**																		
West Coast	-	-	-	-	3	5.4	-	-	-	-	3	8.3	-	-	-	-	0	0.0
Cape Winelands	-	-	-	-	7	12.5	-	-	-	-	7	19.4	-	-	-	-	0	0.0
Overberg	-	-	-	-	3	5.4	-	-	-	-	3	8.3	-	-	-	-	0	0.0
Garden Route	-	-	-	-	1	1.8	-	-	-	-	1	2.8	-	-	-	-	0	0.0
Central Karoo	-	-	-	-	0	0.0	-	-	-	-	0	0.0	-	-	-	-	0	0.0
Cape town Metropole	-	-	-	-	42	75.0	-	-	-	-	22	61.1	-	-	-	-	20	100.0
**Setting type**																		
Clinic	-	-	-	-	31	55.4	-	-	-	-	14	38.9	-	-	-	-	17	85.0
Community day centre	-	-	-	-	18	32.1	-	-	-	-	16	44.4	-	-	-	-	2	10.0
Medical practitioners’ rooms	-	-	-	-	5	8.9	-	-	-	-	5	13.9	-	-	-	-	0	0.0
Other	-	-	-	-	10	17.9	-	-	-	-	9	25.0	-	-	-	-	1	5.0

IQR, interquartile range; HCP, healthcare practitioner; s.d., standard deviation; MBChB, Bachelor of Medicine and Bachelor of Surgery.

† , Not selected job qualification was grouped with nurses, as the clinic confirmed the questionnaire was only distributed to a nursing population.

Other setting types specified included district hospitals (*n* = 4), hospitals (*n* = 4), specified infection clinic at Tygerberg (Ravensmead’s primary referral facility) (*n* = 1) and a private nurse practice (*n* = 1). These participants were included as they were visiting PHC settings while based at hospitals. Participants (*n* = 7) selected more than one setting type.

### Knowledge

The average score of all HCPs in the knowledge domain of the questionnaire was 66.7% (mean: 12.1, s.d.: 4.2) (Table 2). Medical practitioners obtained a statistically significant higher knowledge score compared to nurses (mean difference [MD]: 3.9, *p* = 0.00, 95% CI: 1.82–6.04) (Table 2). In this sample, significantly fewer nursing practitioners were familiar with menopause (*p* = 0.00), obesity (*p* = 0.00) and hysterectomies (*p* = 0.00) as risk factors for UI compared to medical practitioners. Diet (23.2%, *n* = 13), smoking (30.4%, *n* = 17) and family history (41.1%, *n* = 23) were the least selected risk factors by all HCPs (Online Appendix 1 – Addendum N). Medical practitioners were significantly more likely to select pelvic floor muscle training (*p* = 0.01) and medication (*p* = 0.00) as initial UI treatment options (Online Appendix 1 – Addendum O). Significantly fewer nurses in this sample were aware that it is not necessary to refer all female patients with UI to a specialist (*p* = 0.00) compared to medical practitioners.

**TABLE 2:** Physician and nursing practitioners knowledge scores (*n* = 56).

Knowledge questions	Whole population (*n* = 56)						Medical practitioners (*n* = 36)						Nurses (*n* = 20)						*p*	Risk estimate	95% CI
	*n*	%	Mean	s.d.	Median	IQR	*n*	%	Mean	s.d.	Median	IQR	*n*	%	Mean	s.d.	Median	IQR			
**Risk factors for UI**																					
Age	51	91.1	-	-	-	-	34	94.4	-	-	-	-	17	85.0	-	-	-	-	0.34	1.11	0.91–1.36
Pregnancy	48	85.7	-	-	-	-	33	91.7	-	-	-	-	15	75.0	-	-	-	-	0.12	1.22	0.93–1.60
Menopause	35	62.5	-	-	-	-	28	77.8	-	-	-	-	7	35.0	-	-	-	-	0.00*	2.22	1.19–4.14
Hysterectomy	34	60.7	-	-	-	-	29	80.6	-	-	-	-	5	25.0	-	-	-	-	0.00*	3.22	1.48–7.00
Diet	13	23.2	-	-	-	-	9	25.0	-	-	-	-	4	20.0	-	-	-	-	0.75	1.25	0.44–3.55
Diabetes mellitus	40	71.4	-	-	-	-	27	75.0	-	-	-	-	13	65.0	-	-	-	-	0.43	1.15	0.80–1.68
Obesity	43	76.8	-	-	-	-	34	94.4	-	-	-	-	9	45.0	-	-	-	-	0.00*	2.10	1.29–3.43
Urinary tract infection	43	76.8	-	-	-	-	29	80.6	-	-	-	-	14	70.0	-	-	-	-	0.51	1.15	0.83–1.60
Functional or cognitive impairment	38	67.9	-	-	-	-	27	75.0	-	-	-	-	11	55.0	-	-	-	-	0.13	1.36	0.88–2.12
Smoking	17	30.4	-	-	-	-	14	38.9	-	-	-	-	3	15.0	-	-	-	-	0.06	2.59	0.85–7.96
Family history	23	41.1	-	-	-	-	14	38.9	-	-	-	-	9	45.0	-	-	-	-	0.66	0.86	0.46–1.63
Aware that diabetes mellitus has an effect on UI	47	83.9	-	-	-	-	32	88.9	-	-	-	-	15	75.0	-	-	-	-	0.26	1.19	0.90–1.57
Aware that initial UI management can occur in PHC	54	96.4	-	-	-	-	35	97.2	-	-	-	-	19	95.0	-	-	-	-	1.00	1.02	0.91–1.15
**Initial UI treatment options**																					
Pelvic floor muscle training	47	83.9	-	-	-	-	34	94.4	-	-	-	-	13	65.0	-	-	-	-	0.01*	1.45	1.04–2.02
Medication	31	55.4	-	-	-	-	25	69.4	-	-	-	-	6	30.0	-	-	-	-	0.00*	2.32	1.15–4.68
Absorbent products (pads or diapers)	37	66.1	-	-	-	-	25	69.4	-	-	-	-	12	60.0	-	-	-	-	0.47	1.16	0.76–1.76
Bladder training education	47	83.9	-	-	-	-	29	80.6	-	-	-	-	18	90.0	-	-	-	-	0.47	0.90	0.72–1.11
Aware that it is not necessary for all female patients with UI to be referred to a specialist	31	55.4	-	-	-	-	29	80.6	-	-	-	-	2	10.0	-	-	-	-	0.00*	8.06	2.14–30.29
Average knowledge score/18	-	**66.7**	**12.1**	**4.2**	**13.0**	**7.0**	-	**75.2**	**13.53**	**3.4**	**14.0**	**5.0**	-	**53.3**	**9.6**	**4.4**	**9.0**	**6.25**	-	-	-

Note: Bold values are the average of the total scores.

CI, confidence intervals; IQR, interquartile range; PHC, primary healthcare; s.d., standard deviation; UI, urinary incontinence.

* , Indicating a significant result.

Table 2 provides a summary of the knowledge questions included in the questionnaire. Results represent the proportion of the population (%) selecting the correct answer.

### Practice

The average score of all HCPs in the practice domain of the questionnaire was 68.9% (Mean: 9.6, s.d.: 2.4) (Table 3). Medical practitioners had statistically significantly higher practice scores compared to nurses (MD: 2.01, *p* = 0.00, 95% CI: 0.78–3.24) (Table 3), indicating that the practice of medical practitioners in this sample was more congruent with NICE 2013 guidelines. It is concerning to note that only a quarter (25% *n* = 14) of HCPs routinely screened for UI. While 66.7% (*n* = 24) of medical practitioners and 90% (*n* = 18) of nurses were not routinely screening for UI, the reasons for not screening were diverse. Almost half (*n* = 19, 45.2%) of the participants selected the ‘none of the above’ option, indicating that current reasons for not screening patients were invalid for this sample (Table 4). The leading reason for medical practitioners (*n* = 10, 41.7%) was because of a lack of time, while nurses (*n* = 8, 40%) lacked knowledge on how to screen (Table 4). Only 4.8% (*n* = 2) did not screen because of UI being an awkward topic, suggesting that UI was not a taboo topic in this population.

**TABLE 3:** Medical practitioners and nursing practitioners practice scores (*n* = 56).

Practice questions	Whole population (*n* = 56)						Medical practitioners (*n* = 36)						Nurses (*n* = 20)						*p*	Risk estimate	95% CI
	*n*	%	Mean	s.d.	Median	IQR	*n*	%	Mean	s.d.	Median	IQR	*n*	%	Mean	s.d.	Median	IQR			
**Routinely ask if patients have UI**	14	25.0	-	-	-	-	12	33.3	-	-	-	-	2	10.0	-	-	-	-	0.05*	3.33	0.83–13.43
**Asks extent to which UI effect HRQOL**	42	75.0	-	-	-	-	29	80.6	-	-	-	-	13	65.0	-	-	-	-	0.20	1.24	0.87–1.78
**Asks about urinary tract infection if UI is reported**	51	91.1	-	-	-	-	35	97.2	-	-	-	-	16	80.0	-	-	-	-	0.05*	1.22	0.97–1.52
**UI symptoms asked about when UI is reported**																					
Urgency	49	87.5	-	-	-	-	35	97.2	-	-	-	-	14	70.0	-	-	-	-	0.01*	1.39	1.04–1.86
Nocturia	39	69.6	-	-	-	-	27	75.0	-	-	-	-	12	60.0	-	-	-	-	0.24	1.25	0.83–1.87
Urine leakage with effort or exertion or sneezing or coughing	51	91.1	-	-	-	-	36	100.0	-	-	-	-	15	75.0	-	-	-	-	0.00*	1.33	1.04–1.72
Duration of Symptoms	50	89.3	-	-	-	-	36	100.0	-	-	-	-	14	70.0	-	-	-	-	0.00*	1.43	1.07–1.90
Frequency of urinating	47	83.9	-	-	-	-	31	86.1	-	-	-	-	16	80.0	-	-	-	-	0.71	1.08	0.83–1.39
Protective behaviour (pad use)	34	60.7	-	-	-	-	23	63.9	-	-	-	-	11	55.0	-	-	-	-	0.51	1.16	0.73–1.85
Fluid intake daily	36	64.3	-	-	-	-	20	55.6	-	-	-	-	16	80.0	-	-	-	-	0.07	0.69	0.48–1.00
Initiate bladder diaries if UI is reported	8	14.3	-	-	-	-	3	8.3	-	-	-	-	5	25.0	-	-	-	-	0.12	0.33	0.09–1.25
Conduct pelvic assessments if UI is suspected	30	53.6	-	-	-	-	24	66.7	-	-	-	-	6	30.0	-	-	-	-	0.01*	2.22	1.10–4.51
Follow up on patients after initial UI management has started	33	58.9	-	-	-	-	26	72.2	-	-	-	-	7	35.0	-	-	-	-	0.01*	2.06	1.10–3.88
**Refer failed initial management for UI**	56	100.0	-	-	-	-	36	100.0	-	-	-	-	20	100.0	-	-	-	-	-	-	-
**Average practice score/**14	-	**68.9**	**9.6**	**2.4**	**10.0**	**3.0**	-	**74.3**	**10.4**	**2.0**	**10.0**	**2.25**	-	**59.6**	**8.4**	**2.5**	**8.0**	**3.0**	-	-	-

Note: Bold values are the average of the total scores.

CI, confidence intervals; HRQOL, health-related quality of life; IQR, interquartile range; s.d., standard deviation; UI, urinary incontinence.

* , Indicates a significant result.

Bladder diaries are an economic evaluation tool that was rarely (14.3%, *n* = 8) used by HCPs. Predominantly because of a lack of knowledge, as 68.1% (*n* = 32) of the 47 HCPs providing reasons for not initiating bladder diaries stated that they are unfamiliar with them (Table 4).

**TABLE 4a:** Elaborations to medical practitioners and nursing practitioners practice answers (*n* = 56).

Practice: Not routinely asking if female patients have UI because	Whole population (*n* = 42)		Medical practitioners (*n* = 24)		Nurses (*n* = 18)		Knowledge or attitude or belief
	*n*	%	*n*	%	*n*	%	
I don’t have time	10	23.8	10	41.7	0	0.0	Attitude
Female patients have other co-morbidities that are more important to be addressed	9	21.4	7	29.2	2	10.0	Belief
UI is an awkward topic to bring up	2	4.8	1	4.2	1	5.0	Belief
I don’t feel comfortable managing UI	2	4.8	0	0.0	2	10.0	Attitude
I am unsure how to screen for UI	10	23.8	2	8.3	8	40.0	Knowledge
None of the above	19	45.2	11	45.8	8	40.0	-
Not selecting any option	1	2.4	1	4.2	0	0.0	-

UI, urinary incontinence.

**TABLE 4b:** Elaborations to medical practitioners and nursing practitioners practice answers (*n* = 56).

Practice: Don’t initiate bladder diaries because	Whole Population (*n* = 47)		Medical practitioners s (*n* = 33)		Nurses (*n* = 14)		Knowledge or attitude or belief
	*n*	%	*n*	%	*n*	%	
I am unfamiliar with bladder diaries	32	68.1	23	69.7	9	64.3	Knowledge
I don’t have time to initiate bladder diaries	5	10.6	5	15.2	0	0.0	Attitude
Bladder diaries are not necessary for UI evaluation	0	0.0	0	0.0	0	0.0	Knowledge
None of the above	13	27.7	8	24.2	5	35.7	-

UI, urinary incontinence.

**TABLE 4c:** Elaborations to medical practitioners and nursing practitioners practice answers (*n* = 56).

Practice: I don’t conduct pelvic assessments because	Whole Population (*n* = 26)		Medical practitioners (*n* = 12)		Nurses (*n* = 14)		Knowledge or attitude or belief
	*n*	%	*n*	%	*n*	%	
I am unsure how to conduct a pelvic assessment	8	30.8	6	50.0	2	14.3	Knowledge
I am uncomfortable with pelvic assessments	3	11.5	0	0.0	3	21.4	Attitude
Pelvic assessments are too time constraining	2	7.7	2	16.7	0	0.0	Attitude
Pelvic assessments are not necessary for initial evaluation	1	3.8	0	0.0	1	7.1	Knowledge
Not applicable to my practice	6	23.2	0	0.0	6	42.9	Knowledge and attitude
I prefer to refer to someone else	12	46.2	9	75.0	3	21.4	Attitude
None of the above	4	15.4	1	8.3	3	21.4	-

UI, urinary incontinence.

**TABLE 4d:** Elaborations to medical practitioners and nursing practitioners practice answers (*n* = 56).

Practice: I don’t follow up on female patients after initial management has been initiated because	Whole Population (*n* = 23)		Medical practitioners (*n* = 10)		Nurses (*n* = 13)		Knowledge or attitude or belief
	*n*	%	*n*	%	*n*	%	
I don’t have time	0	0.0	0	0.0	0	0.0	Attitude
It is not necessary	0	0.0	0	0.0	0	0.0	Knowledge
It is the patient’s responsibility to follow up	3	13.0	1	10.0	2	15.4	Belief
None of the above	20	87.0	9	90.0	11	84.6	-

UI, urinary incontinence.

During evaluation, medical practitioners were significantly more likely to ask about urinary tract infections (*p* = 0.05), urgency (*p* = 0.01), UI with exertion (*p* = 0.00) and the duration of UI symptoms (*p* = 0.00) (Table 3) (Online Appendix 1 – Addendum P). Medical practitioners were also more likely to conduct pelvic assessments (*p* = 0.01) and follow-up on patients after initial UI management started (*p* = 0.01) (Table 3).

All HCPs referred patients with failed initial UI management to a specialist. Although 12.5% (*n* = 7) indicated that they were uncertain which specialist to refer to. Most referrals were to gynaecologists (48.2%, *n* = 27), urogynaecologists (42.9%, *n* = 24) and urologists (37.5%, *n* = 21), and only 14.3% (*n* = 8) referred to physiotherapists (Online Appendix 1 – Addendum Q). Participants were able to select more than one referral option.

Table 3 provides a summary of the practice questions included in the questionnaire. Results represent the proportion of the population (%) selecting the correct answer.

The questionnaire was designed to explore reasons for HCPs not adhering to NICE 2013 guidelines. Provided options were informed by scoping review data (Van Vuuren et al. 2021), and categorised according to knowledge, attitude or beliefs (Table 4). Healthcare practitioners could select multiple options.

Pelvic assessments were not performed by 33.3% (*n* = 12) of medical practitioners and 70% (*n* = 14) of nurses; 50% (*n* = 6) of the medical practitioners did not conduct pelvic assessment, indicating that they were unsure how to. Healthcare practitioners (*n* = 6, 23.2%) stating that pelvic assessments were not applicable to their practice had a bachelor’s degree in nursing and midwifery (*n* = 3), postgraduate diploma in nursing or midwifery or accoucheur or primary care nursing (*n* = 2) and a higher certificate as an auxiliary nurse (*n* = 1).

Medical practitioners (27.8%, *n* = 10) and nurses (65%, *n* = 13) did not follow up with patients after initiating UI management. It remains unclear why HCPs did not follow up on patients, as 80% (*n* = 20) did not select any of the reasons provided, selecting ‘none of the above’.

Provided answers indicate that HCPs’ attitudes towards UI influence their UI management.

### Belief

Beliefs towards UI management were independently explored with two questions:

- 91.9% (*n* = 51) of HCPs selected false to: ‘It’s not necessary to ask elderly female patients about UI, as it is a normal part of ageing, that nothing can be done for’, while 5.4% (*n* = 3) were unsure whether this is true or false.
- 94.6% (*n* = 53) of HCPs selected true to, ‘Failure to adequately manage UI will have an effect on female patients’ HRQOL’, while 1.8% (*n* = 1) were unsure.

### Attitude

Attitudes towards UI management were independently explored with two specific questions:

- 51.8% (*n* = 29) of HCPs reported they feel comfortable to manage UI.
- 92.9% (*n* = 52) of participants wanted to learn more about UI management.

The underlying reasons for these attitudes were further explored by providing HCPs with previously explored options from a scoping review. Reasons were categorised as knowledge, attitudes or beliefs (Table 5). Healthcare practitioners could select multiple options.

**TABLE 5a:** Elaborations to physicians and nursing practitioners attitude answers (*n* = 56).

Attitude: Not feeling comfortable managing UI because	Whole population (*n* = 27)		Medical practitioner (*n* = 14)		Nurse (*n*= 1 3)		Knowledge or attitude or belief
	*n*	%	*n*	%	*n*	%	
I don’t fully understand what UI management consists of	15	55.6	10	71.4	5	38.5	Knowledge
UI is an awkward topic	1	3.7	0	0.0	1	7.7	Belief
UI does not fall under my scope of practice	5	18.5	2	14.3	3	23.1	Attitude and knowledge
None of the above	6	22.2	2	14.3	4	30.8	-
Not selecting any option	1	3.7	1	7.1	0	0.0	-

UI, urinary incontinence.

**TABLE 5b:** Elaborations to physicians and nursing practitioners attitude answers (*n* = 56).

Attitude: Would not like to learn more about UI management because	Whole Population (*n* = 4)		Medical practitioner (*n* = 3)		Nurse (*n* = 1)		Knowledge or attitude or belief
	*n*	%	*n*	%	*n*	%	
I don’t have time	0	0.0	0	0.0	0	0.0	Attitude
UI does not fall under my scope of practice	1	25.0	1	33.3	0	0.0	Attitude and knowledge
I don’t feel comfortable treating female patients with UI	1	25.0	0	0.0	1	100.0	Attitude
None of the above	1	25.0	1	33.3	0	0.0	-
Not selecting any option	1	25.0	1	33.3	0	0.0	-

UI, urinary incontinence.

Feeling uncomfortable was mainly attributed to a lack of knowledge of what UI management entails for medical practitioners (71.4%, *n* = 10) and nurses (38.5% *n* = 5). Table 5 highlights that discomfort was not because of the sample experiencing UI as an awkward topic (*n* = 1, 3.7%), indicating that UI was not a taboo for this sample. A small minority of HCPs (7.1%, *n* = 4) indicated that they were not interested in learning more about UI management.

### Associations

Associations were explored based on existing evidence. Significant moderate positive associations were found between knowledge and practice scores for this sample’s medical practitioners (*r* = 0.56, *p* = 0.00, *n* = 36). In the nursing sample, a moderate positive association was seen but was not found to be significant (*r* = 0.43, *p* = 0.06, *n* = 20). Associations need to be further explored in an adequate sample size. Results indicate that higher knowledge led to better practice in medical practitioners.

Years in practice were not associated with knowledge (*r* = −0.058, *p* = 0.674) or practice (*r* = 0.058, *p* = 0.676) scores, contradicting other studies. Similarly, despite the importance of UI referral pathways mentioned in other studies, having a UI referral pathway in facilities did not result in statistically significant higher practice scores for HCPs (*p* = 0.367).

When comparing HCPs who were comfortable with UI to those who were uncomfortable, no significant MD was seen in practice scores (MD: 1.02, 95% CI: –2.34–2.29, *p* = 0.11) or knowledge scores (MD: 2.03, 95% CI: –0.17–4.23, *p* = 0.07).

## Discussion

The knowledge and practice of PHC workers in the Western Cape are not in agreement with NICE 2013 standards. While beliefs of medical practitioners and nurses were positive towards UI management, HCPs’ attitudes towards UI management influenced their adherence to EBP.

Healthcare practitioners in this sample lacked a comprehensive knowledge on UI risk factors, predominantly in the nursing population. This finding is similar to other studies (Nguyen, Hunter & Wagg 2013; Witkoś & Hartman-Petrycka 2019), where a lack of knowledge regarding smoking, obesity, diabetes and menopause as UI risk factors among HCPs has been described in various settings. If HCPs are not aware which patients are at risk for UI, they cannot adequately screen patients.

Screening for UI was not routine practice in this sample, also previously reported (Brown et al. 2018; Cooke, O’Sullivan & O’Reilly 2018; Mazloomdoost et al. 2017, 2018; Nguyen et al. 2013; Wagg, Kendall & Bunn 2017; Wong, Kaneshiro & Oyama 2019). Inadequate screening could contribute to the increasing UI prevalence, influencing patients’ HRQOL and contributing to the economic burden of UI. Bladder diaries are an economic evaluation tool, yet they were rarely incorporated into routine practice, also seen in other studies (Coşkun et al. 2017; Jonasson & Josefsson 2016), predominantly attributed to a lack of knowledge regarding bladder diaries. Similarly, physiotherapy is an economic conservative intervention for UI (Vaz et al. 2019). Most HCPs indicated pelvic floor muscle training and bladder training education as an initial UI treatment option. However, few HCPs referred to specialist UI physiotherapists, which was also seen in other studies in South Africa (Padayachey 2009) and internationally (Anger et al. 2016; Mandl, Halfens & Lohrmann 2015; Sinha et al. 2018; Wong et al. 2019). Unnecessary UI referral to a specialist would increase the economic burden, and few nursing HCPs knew that all UI patients did not need to be referred to a specialist. Various studies (Dessie et al. 2015; Jang et al. 2015; Jonasson & Josefsson 2016; Mazloomdoost et al. 2017, 2018; Moskowitz et al. 2018; Sinha et al. 2018; Wong et al. 2019) also noted inadequate UI referrals from HCPs. It is therefore important to address knowledge gaps regarding UI risk factors, bladder diaries and inadequate referrals and ensure that it becomes part of routine practice. Other studies (Mazloomdoost et al. 2017, 2018; Nguyen et al. 2013; Padayachey 2009; Wong et al. 2019) had similar findings, where UI specialist referrals were predominantly to gynaecologists, urogynaecologists and urologists.

Positive attitudes were observed among the majority of HCPs wanting to learn more about UI management, creating an opportunity to incorporate UI education programmes into facilities. Improving UI knowledge could influence attitudes such as ‘not having time to manage UI’, which was prominent among medical practitioners in the sample, as awareness of the importance of UI management could lead to HCPs making time. Viewing UI as too time-consuming was observed in other studies in South Africa (Padayachey 2009) and internationally (Brown et al. 2018; Cooke et al. 2018; Dessie et al. 2015; Ferdinand 2018; Jirscehele et al. 2015; Ostaszkiewicz, O’Connell & Dunning 2016; Wagg et al. 2017). Further, HCPs feeling uncomfortable managing UI were primarily attributed to knowledge, highlighting the need for future intervention on this topic to improve adherence to guidelines. Other studies highlight being uncomfortable with UI management, attributing it to lack of training and support (Nguyen et al. 2013), viewing UI management as an unpleasant experience (Hutchings & Sutherland 2014) and feeling embarrassed regarding UI (Nguyen et al. 2013). A stigma of UI being a sensitive topic was also seen (Cooke et al. 2018; Luo et al. 2016); however, this was not observed in this sample. A study (Padayachey 2009) in South Africa highlighted that GPs had feelings of despair and frustration towards UI discussions, which is hypothesised to influence the initiation of UI conversations.

Healthcare practitioners’ belief that UI is not a normal part of ageing and should be addressed, as well as the belief that UI affects female patients HRQOL, has a positive impact on management. This was contradictory to other studies where UI was believed to be a normal part of ageing (Hälleberg Nyman et al. 2016; Jonasson & Josefsson 2016; Ostaszkiewicz et al. 2016; Ostaszkiewicz, Tomlinson & Hutchinson 2018; Yenişehir, Karakaya & Karakaya 2019). Beliefs that female patients have more important comorbidities to address, that UI is an awkward topic and that it is the patient’s responsibility to follow up were not barriers to UI management but should still be considered when developing an intervention to address UI management as they influenced some HCPs. Healthcare practitioners in this sample knew that UI could be managed at a PHC level; however, UI was not managed at all facilities, indicating a barrier to UI management. In South Africa, all patients in the public sector are seen at a PHC level by a nurse and then, if necessary, by a medical practitioner. Patients can then be referred for further healthcare. A study in South Africa (Padayachey 2009) also highlighted a lack of knowledge regarding UI management for GPs in greater Johannesburg, with little awareness of available UI guidelines. General practitioners in this sample felt UI was beyond their scope of practice, which was also confirmed by a minority of HCPs in our study.

To improve UI management at a PHC level, address the economic burden and decrease the high prevalence of UI, clinical practice must change. Our study highlights the need to address UI knowledge regarding UI risk factors, bladder diaries and how to screen for UI. It is hypothesised that improving UI knowledge could contribute to medical practitioners and nursing practitioners not feeling uncomfortable with UI management and might influence HCPs’ attitude that they do not have time for UI. Participants indicated that they do not conduct pelvic assessments as they prefer to refer to someone else. This can contribute to financial constraints and needs to be explored with qualitative research to understand why medical practitioners and nursing practitioners prefer to refer patients. Further, qualitative research is required to understand why they are not screening for UI, following up on patients, initiating bladder diaries or indicating they do not have time for UI management, to structure an intervention to change clinical practice. Lastly, awareness regarding physiotherapists’ role in conservative UI management needs to be raised to improve referrals. Studies have shown that female patients with UI do not routinely seek help (Jacobs et al. 2017); therefore, our advice should not be generalised to only medical practitioners and nurses in PHC. The multidisciplinary team is able to screen for UI and educate on risk factors, and management available to address UI, and to refer where necessary. Urinary incontinence screening should thus become part of a ‘vital sign’ assessment for any female patient at a PHC level.

Our data must be interpreted with caution. Because of COVID-19 precautions, data collection was completed online, and a snowball sampling method was incorporated, as access to facilities was limited. It is hypothesised that this may limit response rates; however, our results are similar to studies in South Africa and internationally. As questionnaires were not completed by facility managers, a conclusive understanding of facility demographics is not possible. Participants were asked to complete the facility demographic section to the best of their knowledge, and data should therefore be interpreted as the participants’ perception.

Data were predominantly received from medical practitioners and nurses practicing in the Cape Town metropole in the public sector. Therefore, the data should not be generalised to the entire Western Cape population. The questionnaire was developed based on the NICE 2013 (NICE 2013) guideline as well as findings from a scoping review (Van Vuuren et al. 2021). To make the questionnaire user-friendly, it did not include all of the suggestions in the NICE 2013 (NICE 2013) guideline for UI knowledge and practice. Researchers can now use the questionnaire reported in our study to explore the concepts of knowledge, attitudes, beliefs and practices towards UI management in their environment. The pilot questionnaire used in our study did not undergo reliability testing, and reliability testing is recommended for future research before data collection occurs.

## Conclusion

The knowledge and practices of medical practitioners and nursing practitioners working at a PHC level in the Western Cape are not yet in agreement with the NICE 2013 standards. The lack of knowledge regarding UI screening, bladder diaries and UI risk factors needs attention. Whether addressing the knowledge gaps will increase referrals to specialised physiotherapists needs to be investigated. Strategies to ensure routine screening of all women visiting a PHC facility can be developed in consultation with local facilities. The introduction of bladder diaries has the potential to empower women and change their help-seeking behaviour. To ensure a comprehensive team approach to the management of this debilitating condition, the knowledge attitudes, beliefs and practices towards UI of physiotherapists working in PHC must now be investigated.
